# Fecal antibody levels as a noninvasive method for measuring immunity to gastrointestinal nematodes in ecological studies

**DOI:** 10.1002/ece3.1858

**Published:** 2015-12-08

**Authors:** Kathryn A. Watt, Daniel H. Nussey, Rachel Maclellan, Jill G. Pilkington, Tom N. McNeilly

**Affiliations:** ^1^ Institutes of Evolutionary Biology and Immunology and Infection ResearchSchool of Biological SciencesUniversity of EdinburghWest Mains RoadEdinburghUK; ^2^ Moredun Research InstitutePentlands Science ParkBush LoanMidlothianUK

**Keywords:** Fecal egg counts, immunoglobulin, *Ovis aries*, Soay sheep, strongyle nematode, *Teladorsagia circumcincta*

## Abstract

Among‐individual variation in antibody‐associated immunity to gastrointestinal nematode parasites (GIN) is known be associated with life‐history traits and vital rates in wild vertebrate systems. To date, measurement of levels of antibodies against GIN antigens in natural populations has exclusively been based on invasive blood sampling techniques. Previous work in laboratory rodents and ruminant livestock suggests that antibody measures from feces may provide a viable noninvasive approach. We measured total and anti‐GIN antibodies of different isotypes (immunoglobulin (Ig) G, IgA and IgE) from paired samples of plasma and feces from free‐living Soay sheep of different ages and sexes. We tested the correlations among these measures as well as their associations with body mass and Strongyle nematode fecal egg counts (FEC). Significant positive correlations were present among plasma and fecal anti‐GIN antibody levels for IgG and IgA. Generally, correlations between total antibody levels in plasma and feces were weaker and not significant. No significant relationships were found between any antibody measures and body mass; however, fecal anti‐GIN antibody levels were significantly negatively correlated with FEC. Our data clearly demonstrate the feasibility of measuring anti‐GIN antibodies from fecal samples collected in natural populations. Although associations of fecal antibody levels with their plasma counterparts and FEC were relatively weak, the presence of significant correlations in the predicted direction in a relatively small and heterogeneous sample suggests fecal antibody measures could be a useful, noninvasive addition to current eco‐immunological studies.

## Introduction

Establishing how and why immunological variation predicts parasite burden and fitness under natural conditions is a central challenge with the field of ecological immunology (Graham et al. 2011; Schmid‐Hempel 2011). Gastrointestinal nematodes (GIN) are ubiquitous and have important impacts on health in naturally occurring vertebrate populations (Gulland 1992; Albon et al. 2002; Cattadori et al. 2005; Pedersen and Greives 2008). Among‐individual differences in the prevalence and burden of such parasites are therefore likely to have important downstream consequences for life‐history traits and fitness. Variation in immunity to such parasites is a major source of these differences, with protection mediated via antibodies acting at mucosal surfaces being particularly important in the context of GIN (Smith et al. 1985; Gill et al. 1993; Stear et al. 1995; Miquel et al. 2005; McCoy et al. 2008).

Although measurement of antibodies in serum or plasma taken from wild vertebrates as an indicator of immunity or immune investment is increasingly common (e.g., Raberg and Stjernman 2003; Hayward et al. 2014; Ramos et al. 2014), we are not aware of any field studies that have assessed antibodies to GIN from fecal samples, although this approach has been explored in nonfield settings (e.g., Wedrychowicz et al. 1985; Gill et al. 1993). Fecal antibody measures have potential practical advantages for ecologists, as they can be collected noninvasively. They may also provide a clearer immunological signal with respect to GIN as they are more likely to reflect mucosal antibody activity at the site of infection than circulating plasma antibodies (Forrest 1992; Ahren et al. 1998). Here, we measure both plasma and fecal antibodies against a prevalent GIN in a free‐living sheep population and relate these measures to one another and to body mass and parasite fecal egg counts. We show that GIN‐specific antibodies in feces and plasma are positively correlated, and our results suggest that fecal antibody levels represent an overlooked but potentially important tool for ecological immunologists interested in GIN parasite and host interactions and their consequences for evolutionary and population dynamics.

Mucosal antibodies play a central role in the development and maintenance of resistance to GIN (Smith et al. 1985; Stear et al. 1995; Miquel et al. 2005). In mammals, immunoglobulin A (IgA) is by far the most abundant antibody isotype at mucosal surfaces, where it is predominantly produced by plasma cells within the mucosal lamina propria (Brandtzaeg and Johansen 2005; Brandtzaeg 2013). Less abundant antibody isotypes present at mucosal surfaces include IgG, the predominant isotype in serum and the extravascular space, and IgE (Butler 1983; Sasai et al. 1992). In contrast to IgA, IgG within mucosal secretions is largely derived from the circulation (Butler 1983; McNeilly et al. 2007). IgE is primarily produced by plasma cells located in lymph nodes draining sites of antigen entry, or locally at sites of allergic reactions. (Alizadeh et al. 1986; Takhar et al. 2005). With respect to function of these isotypes, there are some notable differences: IgA, unlike IgG, is poor at activating complement and acting as an opsonin and is largely thought to act by binding to and neutralizing invading pathogens (Brandtzaeg 2013; Gutzeit et al. 2014), whereas IgE is uniquely capable of binding to and activating mucosal mast cells which are associated with parasite clearance (Miller and Jarrett 1971).

In relation to GIN infections, IgA and IgE have been studied in the most detail: parasite‐specific IgA and IgE are known to associate with resistance to particular GIN species, with levels of both isotypes being negatively correlated with parasite fecundity and/or numbers (Miller and Jarrett 1971; Sayers et al. 2008); Smith et al. 1985; Stear et al. 1995; Huntley et al. 2001; Strain et al. 2002; Martinez‐Valladares et al. 2005). IgG has also been shown to play a role in reducing GIN burdens in mice (McCoy et al. 2008). How these antibodies work to reduce GIN burdens is unclear, although in sheep IgA has been associated with slower parasite development and reduced fecundity while IgE is associated with reduced mucosal establishment of incoming infective larvae and reduced parasite numbers (Stear et al. 1995).

To study the impact of mucosal antibody responses on GIN populations, it is first necessary to accurately quantify or predict levels of parasite‐specific antibody at the mucosal surface. Techniques involving direct sampling of the mucosal surface at postmortem are the gold standard, but do not allow repeated sampling of the same individual. To overcome this problem, elegant models have been developed in which cannulae are surgically implanted into efferent lymphatic vessels draining the gastrointestinal tract, allowing repeated measurement of antibody levels produced at the site of GIN infection (Smith et al. 1984; Hein et al. 2004). While direct sampling and cannulation‐based techniques have been extremely useful for dissecting the role of mucosal antibodies in GIN immunity, neither are amenable to large scale ecological or population‐based studies. As a result, the majority of such studies have relied on assessment of parasite‐specific antibody levels within serum or plasma, despite some concerns that circulating levels of these antibodies may only partly reflect levels at the mucosal surface (Sinski et al. 1995). Rather surprisingly, few studies have used fecal samples to assess anti‐GIN antibody responses, despite the noninvasive method of collection and the ability to repeatedly sample the same individuals over time. This may partly reflect the lack of data correlating levels of GIN‐specific antibodies within feces with those at either the site of infection or within the circulation, although evidence from other experimental systems would suggest that antibody levels within mucosal, fecal and circulatory compartments are likely to be correlated (Martinez‐Valladares et al. 2005; Nygren et al. 2008).

The Soay sheep (*Ovis aries*) of St Kilda are an unmanaged and unpredated population in which both resistance and tolerance to GIN parasites have shown to be highly variable and under natural selection (Hayward et al. 2011, 2014). *Teladorsagia circumcincta* is a highly prevalent GIN parasite of the Soay sheep (Craig et al. 2006). IgA and pan‐isotype antibodies to *T. circumcincta* measured in plasma have shown negative associations with strongyle fecal egg counts (Coltman et al. 2001; Hayward et al. 2014), while plasma IgG antibodies to this parasite in adults predict over‐winter survival (Nussey et al. 2014). Together these data suggest that variation in circulating anti‐*T. circumcincta* antibodies reflects differences in resistance to the parasite and has important fitness consequences, under conditions of natural infection (Hayward et al. 2014; Nussey et al. 2014). Here, we use paired fecal and plasma samples collected from known individuals in this population to test the utility of fecal antibody levels as noninvasive indicators of plasma levels, as well as the relative ability of both types of measures to predict variation in body mass and strongyle fecal egg counts.

## Materials and Methods

### Fieldwork and sample collection

**Figure 1:**
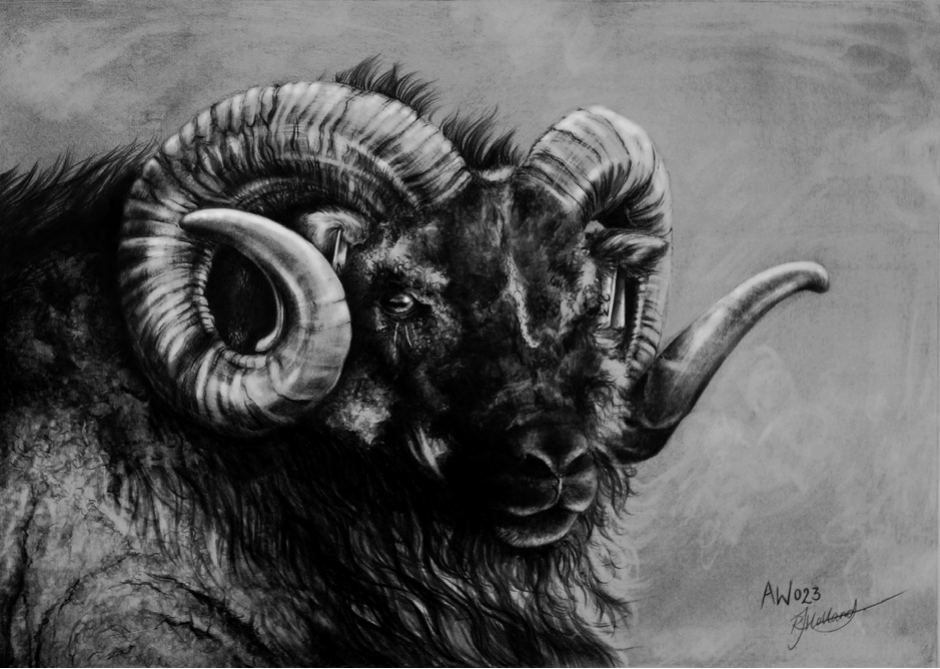
AW023, an extremely successful Soay tup who lived in the Village Bay area of Hirta, St Kilda. Drawing by Rebecca Holland.

All samples were collected in August 2013 from Soay sheep (Fig. 1) from the Village Bay area on the island of Hirta in the St Kilda archipelago. Since 1985, the individuals resident in this area of Hirta have been the subject of long‐term individual‐based monitoring (Clutton‐Brock and Pemberton 2004). All animals selected for sampling had been caught and marked within a few days of birth, and so were of known age and had been monitored throughout their lives. Over two weeks in August 2013, as many sheep from the study population as possible were rounded up in a series of temporary traps, caught, and processed. At capture, individuals were weighed, fecal sampled and whole blood was collected into heparin tubes. Within 24 h of collection, blood samples were centrifuged at 1000 ×  *g* for 10 min and the plasma removed and stored at −20°C.

We selected 50 individuals that were caught in August 2013 for fecal antibody sampling. These comprised 22 lambs (11 females and 11 males), 22 adults aged 2–6 years (15 females and 7 males) and 6 geriatric females aged 7 or more years. Note that very few males live past 6 years, but previous analysis suggests that females show declines in survival probability and immune‐parasitological measures beyond this point (Hayward et al. 2009; Colchero and Clark 2012; Nussey et al. 2012). All fecal samples were collected manually from the rectum at capture. Fecal samples were divided into two subsamples at collection. The first was stored at −20°C for later processing for antibody analysis. The second was used for a strongyle nematode fecal egg count (FEC), which was undertaken typically within two weeks of collection. FEC was estimated as the number of eggs per gram using a modified McMaster technique (following Gulland and Fox 1992). On St Kilda, five nematode species contribute to this count, the most abundant being *Teladorsagia circumcincta*,* Trichostrongylus axei,* and *Trichostrongylus vitrinus* (Craig et al. 2006). FEC has been shown to correlate positively with actual worm burdens counted postmortem, to decline over the first few years of life as immunity develops, and then to increase again in later life (Clutton‐Brock and Pemberton 2004; Hayward et al. 2009). FEC has also been shown to be negatively related to body mass at the time of sampling and to subsequent survival, especially in young animals (Hayward et al. 2011).

### Plasma antibody measures

In the plasma samples, we measured total levels of IgA and IgG and anti‐*T. circumcincta* third larval stage (L3) antibodies for the isotypes IgA, IgG, and IgE, as described previously by Nussey et al. (2014). For total Ig assays, plates were coated overnight at 4°C with 50 *μ*l/well of polyclonal rabbit anti‐ovine IgA or anti‐ovine IgG (AbD Serotec catalogue numbers AHP949 and 5184‐2104 for IgA and IgG, respectively) diluted to 2 *μ*g/mL in 0.06M Carbonate buffer at pH 9.6. For the *T. circumcincta* assays, plates were similarly coated with 50 *μ*l/well of *T. circumcincta* L3 somatic antigen and diluted to 2 *μ*g/mL in 0.06M Carbonate buffer at pH 9.6. L3 somatic antigen was prepared by resuspending *T circumcincta* L3 in PBS (~5 × 10^5^ larvae per mL) in Lysing Matrix D tubes (MP Biomedicals, Santa Ana, CA, USA) and homogenizing in a Precellys® 24 tissue homogenizer (Bertin Technologies, Montigny‐le‐Bretonneux, France). Debris was pelleted by centrifugation at 16,000 ×  *g* at 4°C, and the somatic antigen containing supernatant stored at −80°C prior to use. Total protein concentration of the L3 antigen preparation was estimated using a Pierce™ BCA Protein Assay Kit (Thermo Scientific, Waltham, MA USA).

Following washing of wells three times in TBST (Tris‐buffered saline containing 0.05% Tween‐20 (Sigma‐Aldrich)), 50 *μ*l of an appropriately diluted Soay sheep plasma sample was added to each well and plates incubated at 37°C for 1 h. Sample dilutions were selected by performing doubling dilutions on a set of test plasma samples until ODs reached background levels for each assay and determining the range of dilutions across which ODs decreased linearly for each assay. An appropriate dilution from the upper part of this linear range was then selected for use in the final ELISA protocols. Final dilutions for plasma samples for the ELISAs were as follows: total IgA: 1:6400; total IgG: 1:1,638,400; anti‐*T. circumcincta* IgA (anti‐Tc IgA): 1:50; anti‐*T. circumcinta* IgG (anti‐Tc IgG): 1:12,800; anti‐*T. circumcinta* IgE (anti‐Tc IgE): 1:50.

Plates were then washed five times with TBST before addition of 50 *μ*l/well of the appropriate rabbit anti‐ovine detection antibody conjugated to horseradish peroxidase (HRP) (anti‐ovine IgA‐HRP, anti‐ovine IgG‐HRP: AbD Serotec, catalogue numbers: AHP949P, and 5184‐2504, respectively). For the anti‐*T.circumcincta* IgE assay, 50 *μ*l of anti‐ovine IgE (mouse monoclonal IgG1, clone 2F1) diluted 1:100 in TBST was added to each well, followed by 1‐h incubation at 37°C, five washes with TBST and then addition of 50 *μ*l/well of goat anti‐mouse IgG1‐HRP detection antibody (AbD Serotec catalogue number: STAR132P), diluted to 0.125 *μ*g/mL in TBST. All plates were then incubated at 37°C for 1 h. Following a final wash step in TBST, 100 *μ*l of SureBlue TMB 1‐Component microwell peroxidase substrate (KPL) was added per well and then left to incubate for 5 min in the dark at 37°C. Reactions were then stopped by adding 100 *μ*l 1M HCl, and optical densities (OD) were read immediately at 450 nm using a Thermo Scientific Multiskan GO Spectrophotometer.

Each assay was performed twice on separate ELISA plates. On each of the *T. circumcincta* ELISA plates, two sample‐free wells were included as negative controls, and two wells with 50 *μ*l of dilute gastric lymph fluid from a *T. circumcincta*‐infected domestic sheep were included as positive controls. On the total Ig plates, we included two sample‐free wells as negative controls and purified sheep IgA or IgG (Alpha Diagnostic International: Purified Sheep IgA: ADI 20006‐3, Purified Sheep IgG: ADI 20006‐1, respectively) as positive controls. We then checked the correlation of ODs across duplicate plates and reran both plates if r < 0.80. For subsequent analyses, we took the average OD across the duplicate runs minus the average of the six negative control well ODs across the two plates as our assay measure. Based on serial dilutions of purified sheep IgA and IgG, the detection limits for the total Ig ELISAs were 1.56 *μ*g/mL for IgA and 1.95 ng/mL for IgG.

### Fecal antibody measures

For the fecal samples, we first extracted supernatant solution from the sample for testing and then adapted the plasma protocols to measure the same five antibody levels. We defrosted each sample, then mixed thoroughly and weighed out a minimum of 4 g of sample. To this, we added PBS containing protease Inhibitors (Complete Mini Protease Inhibitor Cocktail tablets, Cat No.:11836153001, Roche, Basel, Switzerland) at a 1:1 ratio with the fecal sample. This was left to stand for 5 min to allow fecal sample to soften, and then, the pellets were broken up and the sample thoroughly mixed. The mixture was allowed to stand for 20 min, to allow inhibition of proteases and extraction of antibodies into the fluid. The mixture was centrifuged at 15,000× *g* for 5 min, and the supernatant removed and put in a fresh tube. This supernatant was then centrifuged at 15,000 ×  *g* for 5 min, and the original mixture was also centrifuged a second time at 15,000× *g* for 5 min. Both supernatants were removed and combined in a fresh tube. This sample was then used in our antibody assays and was considered to be a dilution of 1:2 of the original fecal sample.

We then applied the same ELISA reagents and protocols as used for the plasma assays to these samples. Appropriate dilutions of fecal samples for subsequent ELISAs were selected as previously described for plasma samples, with doubling dilutions on a set of test fecal samples starting at 1:2. Final sample dilutions for fecal antibody ELISAs were as follows: total IgA: 1:32; total IgG: 1:32; anti‐Tc IgA: 1:2; anti‐Tc IgG: 1:2; anti‐Tc IgE: 1:2. In all respects other than sample preparation and dilution, our fecal antibody assays followed the plasma assay protocols described above. For a few of the samples, there was insufficient sample to run all five ELISA measurements: for three samples, we were not able to assay both anti‐Tc IgA and anti‐Tc IgE, and for a further two samples, we could not assay anti anti‐Tc IgE.

### Statistical analyses

Preliminary analysis revealed that fecal anti‐Tc IgE ODs were indistinguishable from background in 31 of 55 samples, with a further 20 within 0.05 OD units of background levels (as measured by the negative control sample) and the remaining 4 samples within 0.15 OD units of background. We concluded that this antibody was undetectable in feces in the vast majority of samples and did not analyze it further. We examined the correlation coefficients and their statistical significance among the four remaining fecal antibody measures, the five plasma antibody measures, weight, and FEC. We also assessed variation in each antibody measure in relation to age and sex group, using ANOVA to test for differences in means among five groups (11 male lambs, 11 female lambs, seven male adults, 15 female adults, and 6 female geriatrics; see above for definitions of these groups). To test the extent to which plasma antibody measures were predicted by fecal antibody measures independently of differences associated with age and sex and other measures, we ran linear models of each plasma antibody variable including age/sex grouping as a five‐level factor and all fecal measures as linear covariates. The significance of each term in the model was assessed by dropping that term from the model and assessing the change in model explanatory power based on F statistics, and we sequentially simplified our models by dropping terms with the lowest *F* statistic until only those significant at the *P* < 0.05 level remained in the final model. Finally, we checked that adding each antibody term back into the final model did not significantly improve the model fit, or alter the estimated effect sizes and significance of the terms left in the final model. To test the degree to which fecal and plasma antibodies independently explained variation in weight and FEC, we ran linear models of these two variables. We log‐transformed FEC having added the minimum nonzero value (100 eggs/gram) to all values to normalize this response variable. To avoid overparamaterization, we first asked whether plasma antibody variation explained weight and FEC by starting with a model including age/sex group and all plasma measures and simplifying the model as above. To the simplified model, we added the four fecal antibody terms and repeated the simplification process. All statistical tests were performed in R version 3.0.2 (R Core Team 2013).

## Results

**Figure 2:**
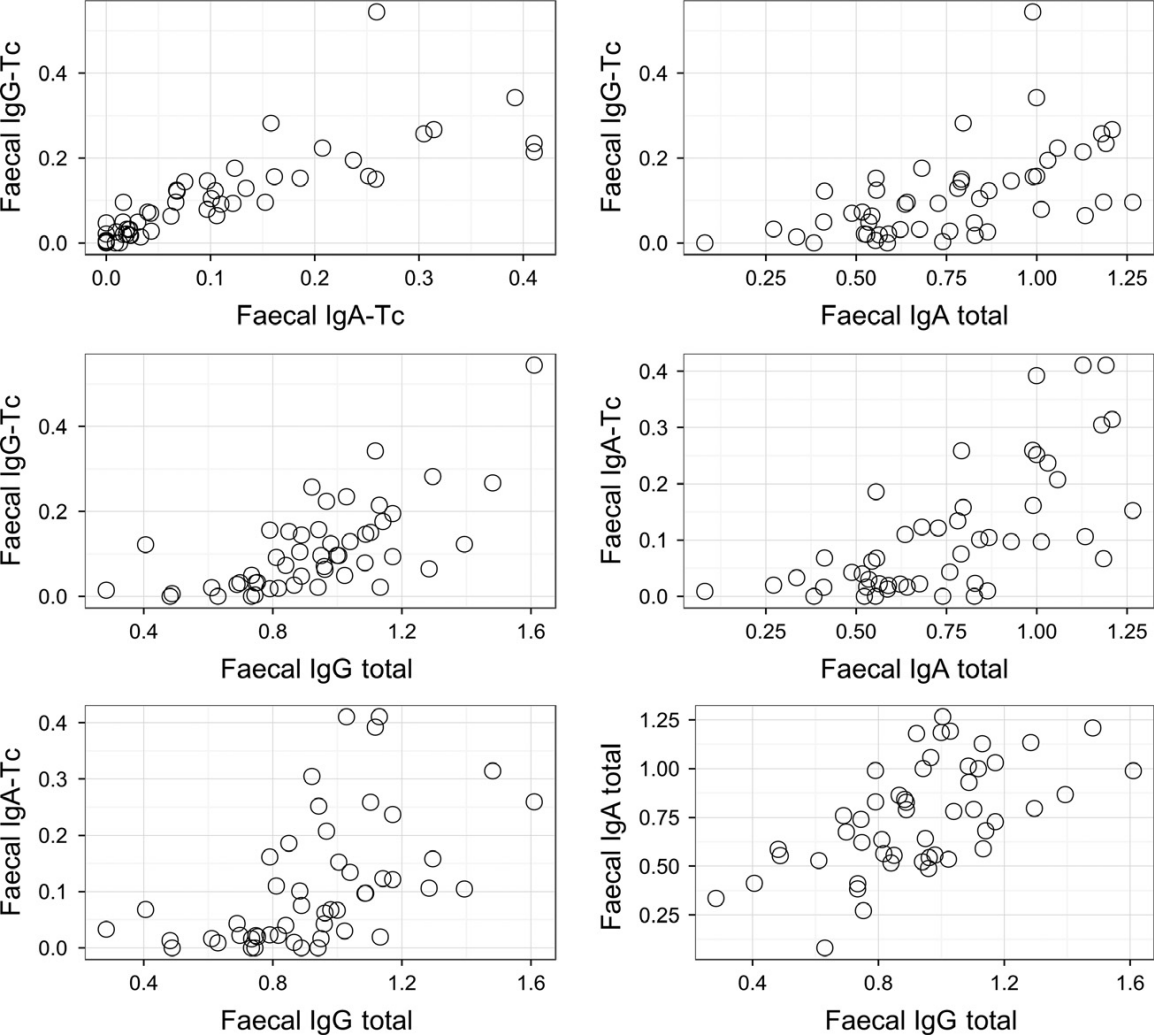
Scatterplots showing relationships among the four fecal antibody measures (“anti‐Tc”: anti‐*T. circumcincta* antibodies). *X*‐axis and *y*‐axis units represent optical densities at a wavelength of 450 nm.

**Figure 3:**
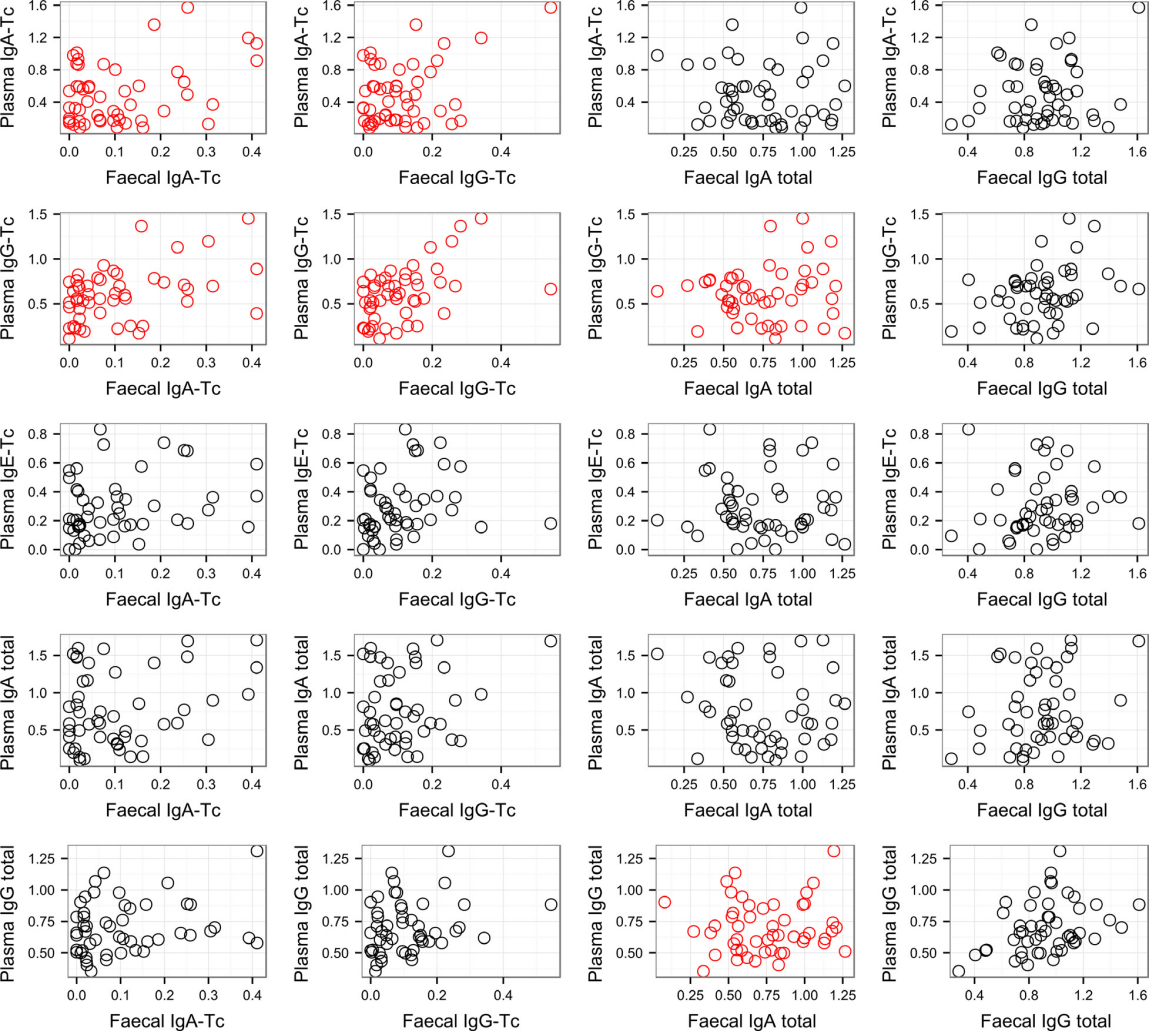
Scatterplots showing relationships between the four fecal antibody measures and the five plasma antibody measures (“anti‐Tc”: anti‐*T. circumcincta* antibodies), with points in red where the correlation was statistically significant (see also Table 1). *X*‐axis and *y*‐axis units represent optical densities at a wavelength of 450 nm.

The four fecal antibody measures (anti‐Tc IgA, anti‐Tc IgG, total IgA, and total IgG) were all significantly positively correlated with one another, with correlations coefficients between 0.54 and 0.81 (Fig. 2, Table S1). Fecal anti‐Tc IgA and IgG antibodies were significantly positively associated with their plasma equivalents (correlation coefficients between 0.35 and 0.49, Fig. 3, Table S1). The only other significant correlation among fecal and plasma measures were between plasma anti‐Tc IgG and total fecal IgA levels and between total plasma IgG and total fecal IgA (0.32 and 0.34, respectively; Fig. 3, Table S1). Fecal anti‐Tc IgA and IgG antibodies showed positive but nonsignificant correlations with weight, and significant negative correlations with FEC (both −0.31, Fig. 5C and D, Table S1). Again, correlations between fecal total antibodies and both weight and FEC were low, nonsignificant, and not consistent in direction (Table S1).

As we have found previously in this population (Nussey et al. 2014), plasma antibody levels were generally positively correlated with one another (coefficients between 0.12 and 0.82, Table S1). All plasma measures were positively correlated with weight and negatively with FEC, with notably high coefficients for anti‐Tc IgE and total IgA (Table S1).

**Figure 4:**
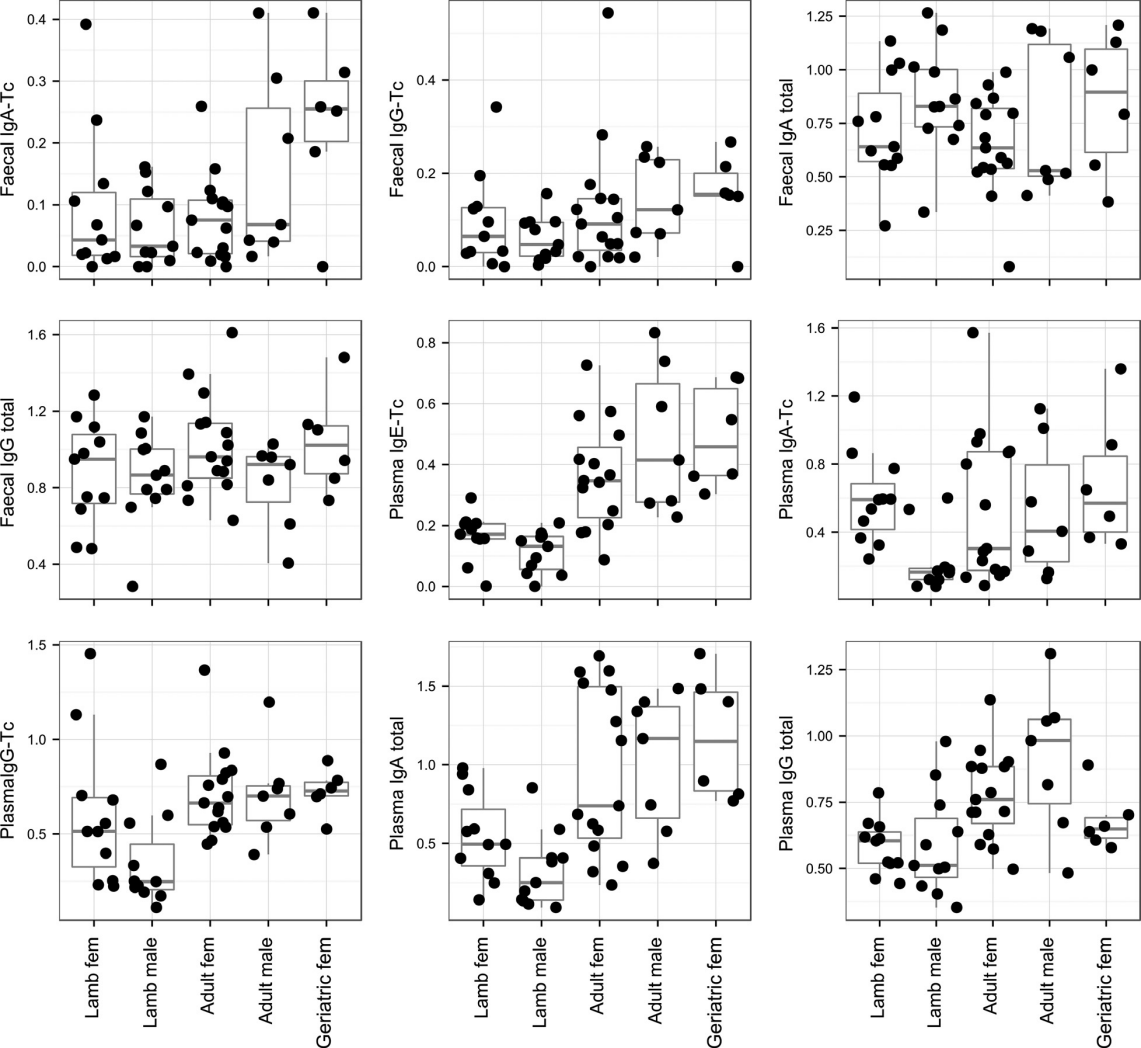
Variation in the four fecal and five plasma antibody measures with respect to age and sex group. Each data point is represented by a black dot. For box plots, the bar in centre of box represents the median value, the box represents the interquartile range, and bars indicate the 5–95th percentile range. fem, female.

Fecal antibody levels showed little evidence of variation among age and sex groups, with only anti‐Tc IgA showing a significant difference among these groups, which was driven by significantly higher levels in female geriatrics compared to all other groups (Fig. 4: anti‐Tc IgA: *F*_(4,45) _= 3.50, *P* < 0.05; anti‐Tc IgG: *F*_(4,45) _= 1.22, *P* = 0.32; total IgA: *F*_(4,45) _= 1.15, *P* = 0.35; total IgG: *F*_(4,45) _= 1.51, *P* = 0.22). This was in contrast to the plasma antibody measures which all showed some form of age or sex dependence, except anti‐Tc IgA levels (Table 1). These were most pronounced for anti‐Tc IgE and total IgA, in which lambs had significantly lower levels than the adult and geriatric groups with no evidence of sex differences (Fig. 4).

**Table 1:** Results from linear models of five plasma antibody measures, with statistical tests (*F* and *P*) and estimated effect sizes and standard errors (b and SE). Terms in bold were retained in the final model following stepwise model simplification: Effect sizes reported are from these final models and statistical tests reflect the change in model explanatory power when a term was dropped from the final model. Terms in plain font were not retained in the final model, and we present statistical significance and estimated effect of the term when added singly back into the final model

	Plasma IgA‐Tc			Plasma IgG‐Tc			Plasma IgE‐Tc			Plasma IgA Total			Plasma IgG Total		
	*F*	*P*	*b* (SE)	*F*	*P*	b (SE)	*F*	*P*	*b* (SE)	*F*	*P*	*b* (SE)	*F*	*P*	*b* (SE)
Age/sex group	0.73	0.57		**3.26**	**<0.05**		**12.01**	**<0.001**		**7.20**	**<0.001**		**6.19**	**<0.001**	
Fecal IgA‐Tc	**8.72**	**<0.01**	**2.11 (0.72)**	**8.50**	**<0.01**	**1.03 (0.35)**	0.01	0.91	0.02 (0.22)	0.20	0.66	0.26 (0.58)	0.12	0.73	−0.10 (0.30)
Fecal IgG‐Tc	1.27	0.27	0.94 (0.83)	1.27	0.27	0.71 (0.63)	0.00	0.98	0.01 (0.22)	0.28	0.60	0.30 (0.58)	1.59	0.21	−0.40 (0.32)
Fecal IgA Total	**4.61**	**<0.05**	**−0.59 (0.27)**	0.05	0.82	0.04 (0.18)	0.08	0.78	0.02 (0.08)	0.20	0.65	−0.10 (0.22)	1.32	0.26	−0.15 (0.13)
Fecal IgG Total	0.29	0.60	0.15 (0.28)	0.99	0.32	−0.21 (0.21)	0.10	0.76	−0.03 (0.09)	0.11	0.74	0.08 (0.24)	**8.26**	**<0.01**	**0.28 (0.10)**

We next tested the ability of different fecal antibody measures to predict each of the five plasma measures independently of each other and of any effects of sex and age (Table 1). Fecal anti‐Tc IgA was a significant, positive predictor of both plasma anti‐Tc IgA and IgG independent of age and sex effects (Table 1). Although fecal anti‐Tc IgG was not significant and was dropped from both these models, when included without fecal anti‐Tc IgA, it was significant (plasma anti‐Tc IgA: *F*_(1,40) _= 7.82, *P* < 0.01, *b* = 1.74 ± 0.62 SE; plasma anti‐Tc IgG: *F*_(1,40) _= 9.44, *P* < 0.01, *b* = 1.08 ± 0.35 SE). This suggests fecal anti‐Tc IgG and IgA are both significant predictor variables, but they explain largely the same variance in plasma anti‐Tc IgG. Fecal anti‐Tc IgA appears to explain very slightly more variance and is hence being retained during model simplification. Total fecal IgA levels significantly negatively predicted plasma anti‐Tc IgA levels, in models accounting for the positive relationship fecal anti‐Tc IgA, and was retained in the final model for this plasma measure (Table 1). Plasma anti‐*T. circumcincta* IgE and total IgA antibodies were not significantly associated with any fecal measures once age and sex effects were accounted for (Table 1). In our models of plasma total IgG including age and sex group, there was a significant positive effect of fecal total IgG, but all other terms were nonsignificant on reintroduction into the final model including only age/sex group (Table 1).

**Figure 5:**
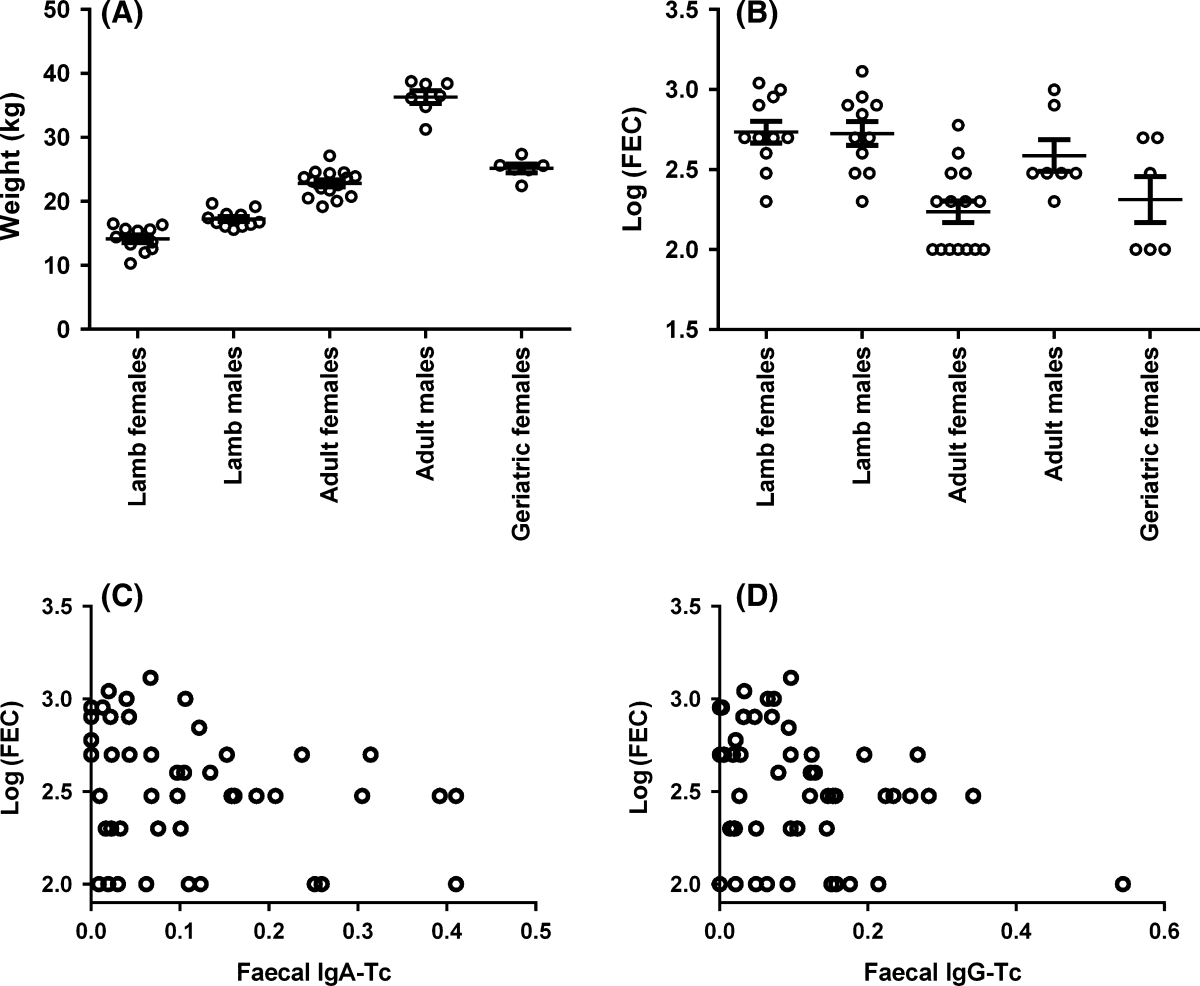
Variation in weight (A) and log‐transformed strongyle fecal egg count (FEC) with respect to age and sex groups, with the mean and SEM indicted by the bars. (C, D): Scatterplot illustrating the significant negative correlation between FEC and fecal anti‐*T. circumcincta* IgA and IgG antibody levels, respectively.

Weight varied very substantially with age and sex, and over 90% of the variation in weight was explained by the age/sex group effect in our models (*r* ^2 ^= 0.93; Table S2, Fig. 5A). Weight was not predicted by any of our antibody measures once age/sex class was accounted for (Table S2). FEC was age and sex dependent (*r* ^2 ^= 0.43; Table S2, Fig. 5B), but after accounting for this fecal anti‐Tc IgA levels significantly negatively predicted FEC (Fig. 5C, Table S2). Total fecal IgG levels were also retained in the final FEC model, showing a positive association with FEC once the negative association with fecal anti‐Tc IgA was accounted for (Table S2). Although fecal anti‐Tc IgG levels were dropped from the final model, they became significant if fecal anti‐Tc IgA was dropped (*F*_(1,40) _= 7.17, *P* < 0.05, *b* = −2.96 ± 1.11 SE). As for our models of plasma anti‐GIN antibodies, this suggests fecal anti‐Tc IgA and IgG are explaining similar variance in FEC but IgA is a slightly stronger predictor of worm burden. None of the other plasma or fecal antibody measures significantly predicted FEC and were dropped from our models (Table S2).

## Discussion

This is the first study, to our knowledge, to examine the utility of fecal antibodies against GIN parasites as an immunological marker in a wild population. We found that significant positive correlations are present between anti‐GIN antibodies measured concurrently in plasma and feces under natural infection conditions. Plasma anti‐GIN IgG antibody levels were found to be similarly positively predicted by either fecal anti‐GIN IgA or IgG once age and sex effects were accounted for. Fecal anti‐GIN IgE was undetectable in this study, possibly as a result of low levels of IgE within the feces and/or low sensitivity of the IgE assays. Depletion of IgG from samples has been shown to increase the sensitivity of IgE measures (Lehrer et al. 2004), presumably by removing any competitive binding of IgG to the allergenic epitopes, and this approach warrants further investigation in future studies. Previous research on our Soay sheep study system has demonstrated important correlations between anti‐GIN antibodies in plasma and FEC and, critically from a fitness perspective, both survival and reproduction in adults (Hayward et al. 2014; Nussey et al. 2014). Given the inherent variability in both infection and immune status present in natural populations of vertebrates and the relative small sample size of the present study, the evident associations between plasma and fecal antibody measures suggest the latter could offer a potentially important noninvasive method to assess GIN resistance and immunity. Once validated as predictors of parasite burden or circulating antibody levels, fecal antibody measures could allow much more intensive repeat sampling of individuals over time in the field, leading to a better understanding of seasonality and longitudinal dynamics of resistance and immunity to GIN in natural conditions.

The plasma antibody measure that was best predicted by fecal measures was anti‐*T.circumcincta* IgG, which we have previously shown to have a strong positive association with over‐winter survival in adult sheep (Nussey et al. 2014). Fecal anti‐Tc IgA and IgG levels both appeared to predict this plasma measure, although they only explained a moderate amount of variation, and appeared to explain largely the same variation such that neither was significant when both were included in the model. This represents the first indication from a natural population that anti‐GIN antibody levels in feces might reflect variation in circulating antibodies against the same parasites.

Positive associations between antibody measurements at mucosal surfaces and in circulation have previously been detected in a variety different host–parasite systems. For instance, parasite‐specific IgA measured in serum and gastric mucus were shown to be highly correlated in domestic sheep experimentally or naturally infected with *T. circumcinca* (Sinski et al. 1995; Martinez‐Valladares et al. 2005). Possessive associations between cholera‐toxin (CT) antigen‐specific circulating and mucosal antibody levels have also been reported in mice mucosally immunized with CT (Externest et al. 2000). In natural populations, sampling at the mucosal surface either postmortem or via cannulization is likely to be both highly impracticable and undesirable. The use of fecal antibodies as a proxy for direct mucosal sampling offers a potentially exciting noninvasive alternative. However, if used as a sole indicator of anti‐GIN immunity, this rests on the assumption that antibody levels within all three (circulatory, mucosal, and fecal) compartments are closely correlated. There is some support for this from rodent mucosal immunization models. For example, intestinal and fecal lipopolysaccharide‐specific IgA were highly correlated in mice mucosally immunized with Vibrio cholera LPS (Nygren et al. 2008), and similar associations were seen between intestinal lavage and fecal CT‐specific IgA in mice immunized orally or rectally with CT (Grewal et al. 2000). That said, parasite‐specific antibodies are known to be sequestered from the circulation via binding to the parasites at the mucosal surfaces (De Cisneros et al. 2014), which could reduce circulating anti‐GIN antibody levels as a function of parasite burden, and could potentially weaken associations between mucosal and circulating antibody levels.

It is important to note that fecal anti‐GIN antibodies could tell us a great deal more about immunity and resistance than a simple reflection of circulating antibody concentrations. They could also indicate important immune activity at the mucosal surfaces which is not detectable in plasma or serum antibody levels and thus may have important independent associations with resistance and fitness. This is exemplified in a previous study in domestic sheep in which GIN‐specific antibody levels in feces showed a stronger negative correlation with FEC compared to antibodies measured in serum (Gill et al. 1993). In the present study, we also found negative associations between anti‐*T. circumcincta* fecal antibody measures, but not their plasma equivalents, and FEC. Previous studies of FEC in the Soay sheep system have demonstrated a population‐wide negative association between FEC and a pan‐isotype anti‐Tc antibody measure in plasma (Hayward et al. 2014) and a negative association between plasma anti‐Tc IgA levels and FEC in young animals (Coltman et al. 2001). However, a recent study of adult females found no clear cut negative associations with plasma anti‐Tc IgA, IgE, or IgG levels in the Soays (Nussey et al. 2014). This suggests the relationship between antibody levels and FEC may be complex and age‐dependent. In addition, it should be noted that *T. circumcincta* is not the only endemic GIN in the Soay population (Craig et al. 2006), and that other GIN species present on St. Kilda may contribute to FEC and host fitness measures. Further study with a larger sample size including different age groups, and measuring antibody responses to other GIN species present on St. Kilda, will be required to test how well fecal anti‐GIN antibody levels predict FEC and fitness outcomes like survival and fecundity independently of plasma levels.

In summary, our data demonstrate the feasibility of measuring anti‐GIN antibody responses using fecal samples in wild animals. Fecal antibody measures may be useful either as markers of circulating levels of antibody and/or independent predictors of resistance to GIN parasites. However, the wider application of fecal antibody measures would still require careful development within any new study system: not all fecal antibody measures will necessarily correlate with plasma antibodies or parasitological measures, and this is likely to vary depending on the host species and parasite studied. A useful first step to understanding the utility of fecal antibodies as a marker of immunity, as performed here, would be to test associations among antibodies from plasma or serum samples and measurements of parasite burdens. Our data suggest that anti‐GIN fecal antibody measurements offer a potentially useful, and thus far overlooked, means for eco‐immunologists to take regular longitudinal immune measurements without the need for invasive sampling techniques.

## Data Accessibility

We will make all data used in this study available via Dryad once the manuscript is acceptable for publication.

## Conflict of Interest

None declared.

## Supporting information


**Table S1.** Pearson's correlation coefficients (significant coefficients at *P* < 0.05 level in bold) among antibody measures, weight and nematode faecal egg counts (FEC) in 50 Soay sheep.
**Table S2.** Results from linear models of weight and log‐transformed strongyle nematode faecal egg count (FEC), with statistical tests (F and P) and estimated effect sizes and standard errors (b and SE).
