# Supplementary material for: Fecal antibody levels as a noninvasive method for measuring immunity to gastrointestinal nematodes in ecological studies
Source: Ecol Evol. 2015 Dec 8;6(1):56–67. doi: 10.1002/ece3.1858 (PMC4716500; doi:10.1002/ece3.1858)
Supplement: Supplementary file 1 — Table S1. Pearson's correlation coefficients (significant coefficients at P < 0.05 level in bold) among antibody measures, weight and nematode faecal egg counts (FEC) in 50 Soay sheep. Table S2. Results from linear models of weight and log‐transformed strongyle nematode faecal egg count (FEC), with statistical tests (F and P) and estimated effect sizes and standard errors (b and SE). [file ECE3-6-056-s001.docx]

**Table S1.** Pearson’s correlation coefficients (significant coefficients at P<0.05 level in bold) among antibody measures, weight and nematode faecal egg counts (FEC) in 50 Soay sheep.

|  |  | Faecal antibody | | | | Plasma antibody | | | | |  | |
| --- | --- | --- | --- | --- | --- | --- | --- | --- | --- | --- | --- | --- |
|  |  | IgG-Tc | IgA-Tc | IgG Total | IgA Total | IgE-Tc | IgG-Tc | IgA-Tc | IgA Total | IgG Total | Weight | FEC |
| Faecal antibody | IgG-Tc |  |  |  |  |  |  |  |  |  |  |  |
|  | IgA-Tc | **0.805** |  |  |  |  |  |  |  |  |  |  |
|  | IgG Total | **0.563** | **0.679** |  |  |  |  |  |  |  |  |  |
|  | IgA Total | **0.661** | **0.536** | **0.592** |  |  |  |  |  |  |  |  |
| Plasma antibody | IgE-Tc | 0.219 | 0.262 | -0.024 | 0.089 |  |  |  |  |  |  |  |
|  | IgG-Tc | **0.490** | **0.431** | 0.080 | **0.322** | **0.390** |  |  |  |  |  |  |
|  | IgA-Tc | **0.375** | **0.350** | -0.046 | 0.167 | 0.122 | **0.305** |  |  |  |  |  |
|  | IgA Total | 0.250 | 0.252 | -0.122 | 0.174 | **0.406** | **0.318** | **0.797** |  |  |  |  |
|  | IgG Total | 0.238 | 0.226 | 0.069 | **0.338** | **0.352** | **0.337** | 0.234 | **0.370** |  |  |  |
|  | Weight | 0.225 | 0.261 | -0.048 | -0.048 | **0.573** | 0.264 | 0.141 | **0.452** | **0.489** |  |  |
|  | FEC | **-0.313** | **-0.310** | 0.036 | -0.106 | **-0.431** | -0.278 | -0.265 | **-0.343** | -0.149 | -0.241 |  |

**Table S2.** Results from linear models of weight and log-transformed strongyle nematode faecal egg count (FEC), with statistical tests (F and P) and estimated effect sizes and standard errors (b and SE). Terms in bold were retained in the final model following stepwise model simplification: effect sizes reported are from these final models and statistical tests reflect the change in model explanatory power when a term was dropped from the final model. Terms in plain font were not retained in the final model and we present statistical significance and estimated effect of the term when added singly back into the final model.

|  | Weight | | | FEC | | |
| --- | --- | --- | --- | --- | --- | --- |
|  | F | P | b (SE) | F | P | b (SE) |
| Age/sex group | **156.00** | **<0.001** |  | **12.40** | **<0.001** |  |
| Plasma IgA-Tc | 1.33 | 0.25 | 0.97 (0.84) | 1.19 | 0.28 | -0.26 (0.24) |
| Plasma IgG-Tc | 0.04 | 0.85 | 0.22 (1.11) | 0.40 | 0.53 | 0.21 (0.33) |
| Plasma IgE-Tc | 0.39 | 0.53 | -1.23 (1.96) | 0.51 | 0.48 | -0.38 (0.54) |
| Plasma IgA Total | 0.29 | 0.59 | 0.40 (0.74) | 1.57 | 0.22 | -0.25 (0.20) |
| Plasma IgG Total | 0.44 | 0.51 | -1.11 (1.68) | 0.36 | 0.55 | -0.30 (0.50) |
| Faecal IgA-Tc | 0.07 | 0.80 | 0.73 (2.85) | **12.17** | **<0.01** | **-3.40 (0.97)** |
| Faecal IgG-Tc | 0.03 | 0.86 | 0.50 (2.86) | 0.20 | 0.66 | -0.67 (1.53) |
| Faecal IgA Total | 1.19 | 0.28 | -1.18 (1.08) | 1.21 | 0.28 | 0.58 (0.52) |
| Faecal IgG Total | 2.23 | 0.14 | -1.70 (1.14) | **7.61** | **<0.01** | **1.10 (0.40)** |
